# Revision of clinical case definitions: influenza-like illness and severe acute respiratory infection

**DOI:** 10.2471/BLT.17.194514

**Published:** 2017-11-27

**Authors:** Julia Fitzner, Saba Qasmieh, Anthony Wayne Mounts, Burmaa Alexander, Terry Besselaar, Sylvie Briand, Caroline Brown, Seth Clark, Erica Dueger, Diane Gross, Siri Hauge, Siddhivinayak Hirve, Pernille Jorgensen, Mark A Katz, Ali Mafi, Mamunur Malik, Margaret McCarron, Tamara Meerhoff, Yuichiro Mori, Joshua Mott, Maria Teresa da Costa Olivera, Justin R Ortiz, Rakhee Palekar, Helena Rebelo-de-Andrade, Loes Soetens, Ali Ahmed Yahaya, Wenqing Zhang, Katelijn Vandemaele

**Affiliations:** a Infectious Hazard Management, World Health Organization, avenue Appia 20, 1211 Geneva 27, Switzerland.; b National Centre for Communicable Diseases, Ulaanbataar, Mongolia.; c Regional Office for Europe, World Health Organization, Copenhagen, Denmark.; d Western Pacific Regional Office, World Health Organization, Manila, Philippines.; e Department of Infectious Disease Epidemiology, Norwegian Institute of Public Health, Oslo, Norway.; f Centers for Disease Control and Prevention, Atlanta, United States of America (USA).; g Eastern Mediterranean Regional Office, World Health Organization, Cairo, Egypt.; h Department of Primary and Community Care, Radboud University Medical Center, Nijmegen, Netherlands.; i United States Centers for Disease Control and Prevention Kenya Office, Nairobi, Kenya.; j Municipal Health Secretariat, Belo Horizonte, Brazil.; k Pan American Health Organization, World Health Organization, Washington, USA.; l Department of Infectious Diseases, National Institute of Health, Lisbon, Portugal.; m Centre for Infectious Disease Control, National Institute for Public Health and the Environment, Bilthoven, Netherlands.; n Regional Office for Africa, World Health Organization, Brazzaville, Congo.

## Abstract

The formulation of accurate clinical case definitions is an integral part of an effective process of public health surveillance. Although such definitions should, ideally, be based on a standardized and fixed collection of defining criteria, they often require revision to reflect new knowledge of the condition involved and improvements in diagnostic testing. Optimal case definitions also need to have a balance of sensitivity and specificity that reflects their intended use. After the 2009–2010 H1N1 influenza pandemic, the World Health Organization (WHO) initiated a technical consultation on global influenza surveillance. This prompted improvements in the sensitivity and specificity of the case definition for influenza – i.e. a respiratory disease that lacks uniquely defining symptomology. The revision process not only modified the definition of influenza-like illness, to include a simplified list of the criteria shown to be most predictive of influenza infection, but also clarified the language used for the definition, to enhance interpretability. To capture severe cases of influenza that required hospitalization, a new case definition was also developed for severe acute respiratory infection in all age groups. The new definitions have been found to capture more cases without compromising specificity. Despite the challenge still posed in the clinical separation of influenza from other respiratory infections, the global use of the new WHO case definitions should help determine global trends in the characteristics and transmission of influenza viruses and the associated disease burden.

## Résumé

La formulation de définitions précises de cas cliniques fait partie intégrante d'un processus efficace de surveillance de la santé publique. Alors que ces définitions devraient, dans l'idéal, s'appuyer sur un ensemble standardisé et fixe de critères de définition, elles nécessitent souvent une révision pour tenir compte des nouvelles connaissances relatives à la maladie concernée et des améliorations apportées aux tests diagnostiques. Pour être optimales, les définitions de cas doivent aussi établir un équilibre entre sensibilité et spécificité qui reflète leur utilisation aux fins prévues. À la suite de la pandémie de grippe H1N1 de 2009-2010, l'Organisation mondiale de la Santé (OMS) a lancé une consultation technique sur la surveillance mondiale de la grippe. Cela a conduit à des améliorations concernant la sensibilité et la spécificité de la définition de cas pour la grippe – c'est-à-dire une maladie respiratoire dont seule la symptomatologie reste à définir. Le processus de révision n'a pas seulement modifié la définition du syndrome de type grippal pour inclure une liste simplifiée des critères le mieux à même de prédire une infection grippale, il a également permis de clarifier le langage utilisé dans la définition pour en améliorer l'interprétation. Par ailleurs, afin de tenir compte des cas sévères de grippe qui nécessitaient une hospitalisation, une nouvelle définition de cas a été introduite concernant l'infection aigüe sévère des voies respiratoires dans tous les groupes d'âge. Il a été constaté que les nouvelles définitions reflétaient davantage de cas, sans pour autant compromettre la spécificité. S'il est vrai que la distinction clinique de la grippe des autres infections respiratoires continue de poser problème, l'utilisation mondiale des nouvelles définitions de cas de l'OMS devrait permettre de dégager des tendances mondiales concernant les caractéristiques et la transmission des virus grippaux ainsi que la charge de morbidité qui leur est associée.

## Resumen

La elaboración de definiciones precisas de los casos clínicos es una parte fundamental de un proceso efectivo de la vigilancia de la salud pública. Aunque tales definiciones deberían, idealmente, estar basadas en una recopilación estandarizada y fija de criterios de definición, a menudo necesitan una revisión para reflejar el nuevo conocimiento de la enfermedad existente y las mejoras en las pruebas de diagnóstico. Las definiciones óptimas de los casos también deben tener un equilibrio entre sensibilidad y especificidad que refleje su uso previsto. Después de la pandemia de gripe H1N1 en 2009-2010, la Organización Mundial de la Salud (OMS) inició una consulta técnica para la vigilancia mundial de la gripe. Esto dio lugar a mejoras en la sensibilidad y la especificidad de las definiciones de los casos de gripe, es decir, una enfermedad respiratoria que carece de una sintomatología definitoria singular. El proceso de revisión no solo modificó la definición de las enfermedades similares a la gripe para incluir una lista simplificada de los criterios que demostraron ser más predictivos de la infección por gripe, sino que también aclaró el lenguaje utilizado para la definición, con el fin de mejorar su interpretación. Para englobar los casos graves de gripe que requirieron hospitalización, también se desarrolló una nueva definición de los casos de la infección respiratoria aguda grave en todos los grupos de edad. Se ha descubierto que las nuevas definiciones engloban más casos sin comprometer la especificidad. A pesar del desafío que todavía plantea la separación clínica de la gripe de otras infecciones respiratorias, el uso global de las nuevas definiciones de los casos de la OMS debería ayudar a determinar las tendencias mundiales en las características y transmisión de los virus de la gripe y la carga de la enfermedad asociada.

## ملخص

إن صياغة تعريفات دقيقة للحالات من الناحية العلاجية جزء أساسي من الإجراءات الفعالة لمراقبة الصحة العامة. وبالرغم من أن مثل هذه التعريفات يجب، من الناحية النظرية، أن تقوم على مجموعة منتقاة من المعايير التعريفية الموحدة والثابتة، فغالبًا ما تحتاج تلك التعريفات إلى المراجعة لكي تعكس المعلومات التي تستجد بشأن الحالة المرتبطة بها والتطورات التي تشهدها الاختبارات التشخيصية. وتحتاج أيضًا التعريفات المثالية للحالات إلى الموازنة بين الحساسية والنوعية بما يتفق مع الاستخدام المزمع. وبعد تفشي فيروس الأنفلونزا **H1N1 ** في الفترة ما بين عامي 2009 و2010، بدأت منظمة الصحة العالمية في تقديم المشاورة التقنية بشأن مراقبة الأنفلونزا على المستوى العالمي. وقد شجع ذلك على تحسين الحساسية والنوعية المرتبطة بتعريف حالة الأنفلونزا - بعبارة أخرى، مرض يصيب الجهاز التنفسي يفتقر إلى مجموعة أعراض فريدة تعرّف المرض. وأدت إجراءات المراجعة إلى تعديل تعريف ذلك المرض المشابه للأنفلونزا، إذ شمل التعريف قائمة مبسطة من المعايير التي تبين أنها الأفضل للتنبؤ بعدوى الأنفلونزا، ولم تقتصر النتائج على ذلك فحسب، بل نتج أيضًا عن تلك الإجراءات استخدام لغة أكثر وضوحًا في التعريف، لتحسين القدرة على التأويل. ولتحقيق الدقة في رصد الحالات الحرجة للأنفلونزا والتي تتطلب الإقامة بالمستشفى، تم أيضًا وضع تعريف جديد لحالة العدوى التنفسية الحادة والحرجة لجميع الفئات العمرية. وتبين أن التعريفات الجديدة ترصد بدقة مزيدًا من الحالات دون الإخلال بالنوعية. وبالرغم من أن التحدي لا يزال قائمًا عند التمييز من الناحية العلاجية بين الأنفلونزا وغيرها من أنواع عدوى الجهاز التنفسي، من المفترض أن يساعد الاستخدام العالمي لما توصلت إليه منظمة الصحة العالمية من تعريفات جديدة للحالة على تحديد الاتجاهات العامة فيما يتعلق بخصائص فيروسات الأنفلونزا وطرق انتقال العدوى بها وكذلك عبء المرض المرتبط بها.

## 摘要

准确制定临床病例定义是进行有效公共卫生监督进程中的一个组成部分。虽然理论上此类定义应该以标准化和固定收集的定义标准为基础，但是这些定义通常需要进行修订，以反映所涉及的新症状知识，以及诊断测试的进展。最佳病例定义还需要达到反映其预期用途敏感性与特异性的平衡。2009–2010 年 H1N1 流感大流行之后，世界卫生组织发起了一项有关全球流感监测的技术咨询。该咨询提出改进流感定义的敏感性与特异性，即呼吸道疾病缺乏独特定义的症状。修订过程不仅修改流感样疾病定义，以纳入显示最能预测流感病毒感染的简化标准列表，同时明确了定义使用的语言，以增强解释力。为了获取需要住院治疗的严重流感病例，同时为所有年龄段的严重急性呼吸道感染制定了新的病例定义。目前已经发现新定义能够囊括更多案例，并且不会影响特异性。尽管在临床上将流感与其他呼吸道感染区分开来仍然面临挑战，但是统一使用世界卫生组织的新病例定义有助于确定全球流感病毒的特征和传播趋势以及相关的疾病负担。

## Резюме

Формулировка точных определений клинических случаев является неотъемлемой частью эффективного эпиднадзора. Хотя в идеале такие определения должны основываться на стандартизованном и фиксированном наборе определяющих критериев, они часто требуют пересмотра с учетом новых знаний об этих состояниях и улучшениях в диагностическом тестировании. Оптимальные определения случаев также должны иметь баланс чувствительности и специфичности, который отражает их целевое назначение. После пандемии гриппа H1N1 в 2009–2010 годах Всемирная организация здравоохранения (ВОЗ) инициировала техническую консультацию по глобальному эпиднадзору за гриппом. Это привело к улучшению чувствительности и специфичности определения для гриппа, то есть респираторного заболевания, которое не имеет однозначной определяющей симптоматики. Пересмотр привел не только к изменению определения гриппоподобного заболевания, включив в него упрощенный список критериев, показавших, что гриппозная инфекция является наиболее прогностической, но и для улучшения интерпретируемости был сделан более понятным язык, используемый для этого определения. Для выявления тяжелых случаев гриппа, требующих госпитализации, было разработано новое определение для тяжелой острой респираторной инфекции во всех возрастных группах. Было обнаружено, что новые определения охватывают больше случаев без ущерба для специфичности. Несмотря на сложности, которые все еще возникают при дифференциации гриппа от других респираторных инфекций, глобальное использование новых определений ВОЗ должно помочь выявить всеобщие тенденции в характеристиках и передаче вируса гриппа, а также в связанном с ними бремени болезней.

## Introduction

Case definitions form an important part of standardized systems for public health surveillance. They can be used in the reporting of the conditions under surveillance, the monitoring of geographical and temporal variations in prevalence, the detection of the increases in incidence that may be indicative of outbreaks and the evaluation of the effectiveness of control and response efforts. By permitting valid comparisons across time and between data from different locations, the use of standard case definitions can improve the usefulness of data generated by a surveillance system.^1^ In general, case definitions for infectious diseases need to be concise and uncomplicated if they are to be useful for, and useable by, all the health-care providers, laboratory staff and public health personnel involved in case reporting.

The usefulness of a case definition depends on the characteristics of the condition of interest, the characteristics of other conditions with which the condition of interest may be confused and the objectives of any surveillance on the condition of interest. Ideally, a case definition would be based on a combination of signs, symptoms and results of laboratory tests that uniquely characterizes the condition of interest. The definition would also be easily applicable. Unfortunately, ease of use tends to decrease as specificity increases and there often needs to be a trade-off between the sensitivity of a case definition and its specificity. The importance of a definition’s specificity, relative to its sensitivity, depends on the definition’s intended use. For example, sensitivity may be particularly important in evaluating the size of an outbreak, when the number of missed cases needs to be minimized. In research, however, it may be more important to minimize the number of false positives enrolled by using a case definition that is particularly specific.

Although a fixed case definition can facilitate valid comparisons of disease occurrence over long time periods, case definitions may often be revised to improve their sensitivity and/or specificity.^1^ The relative values of the clinical, epidemiological and/or laboratory criteria used for case definitions may change over time. For example, an emerging new pathogen could prompt multiple revisions of a case definition.^2^^,^^3^ Further, case definitions have also been modified to enhance their sensitivity across a wider age range of individuals^4^ or to make use of novel diagnostic tests that improve the detection of the disease-causing pathogen.^5^^,^^6^ In other instances, the language used in a case definition has been simplified and/or an attempt has been made to reduce multiple definitions for the same condition to a single global one.^7^ The case definitions for infection with the human immunodeficiency virus and acquired immunodeficiency syndrome were revised multiple times between 1982 and 2014, to reflect new knowledge about the syndrome and the isolation of the virus and to include laboratory testing.^8^

In the revision of any case definition there needs to be careful deliberation over how the available relevant evidence can help balance the sensitivity and specificity of the definition in such a way that the objectives of any related surveillance can be met. In 2011, the World Health Organization (WHO) launched an initiative to develop global standards for influenza surveillance, including a global case definition of severe influenza. This initiative was, in part, a response to the 2009–2010 H1N1 influenza pandemic. Below, as an illustration of case definition revision, we discuss the process followed in revising WHO’s case definitions for both influenza-like illness (ILI) and severe acute respiratory infection (SARI).

## Impetus for change

Since 1999, WHO has recommended a case definition for ILI that can be used for public health surveillance, to monitor the seasonal variations of influenza activity and to identify the best patients to be included in virological surveillance.^9^ The 2009–2010 H1N1 pandemic, however, prompted the further evaluation of the objectives of influenza surveillance in and after 2010 and the examination of whether the case definitions existing in 2010 could be improved to strengthen the usefulness of the surveillance framework and pandemic-preparedness efforts. The appearance of human infections with novel influenza viruses further supported the need to monitor severe influenza-related diseases and understand influenza-associated disease severity and burden more fully. Prior to the 2009–2010 pandemic, no global standard for SARI had been developed for wide implementation. The lack of standardization in case definitions and lack of a systematic approach to the surveillance of mild and severe influenza greatly hampered efforts to monitor and understand the burden and severity of the 2009–2010 pandemic in the global context.

A global standardized case definition for influenza is essential if we are to make valid comparisons of surveillance data collected in different areas of the world. To be very useful in surveillance, an ILI case definition needs to be based on simple criteria, yet have levels of sensitivity and specificity that permit accurate estimates of influenza disease burden and permit influenza to be distinguished from other respiratory illnesses.^10^ The largest challenge for influenza surveillance is that the symptoms of influenza are nonspecific.

A conclusion of several evaluations of the specificity of WHO’s 1999 clinical case definition for influenza was that the definition needed to be revised to enhance its specificity for influenza detection.^11^^–^^14^ In 2010, only 31 of the 105 countries that participated in a WHO survey of national influenza centres reported using this definition.^15^ An earlier investigation of influenza surveillance across western Europe had revealed that case definitions varied between countries.^16^

In a global consultation in 2011, WHO outlined how influenza surveillance should allow influenza seasonality to be evaluated in each country and allow the start of the influenza season to be announced. Surveillance should also establish and monitor baseline trends in ILI and SARI, so that annual changes in severity can be followed and provide data that can be used to understand disease burden and the impact of influenza in relation to other diseases. In addition, it should identify and monitor the groups at high risk for severe disease, so that priorities for the use of resources can be set.^17^ WHO specified that influenza surveillance should also be used to provide virological data and so help in: (i) identifying locally circulating types and subtypes of influenza viruses and their relations to global and regional patterns; (ii) describing the antigenic character and genetic makeup of circulating influenza viruses; (iii) monitoring antiviral sensitivity; (iv) understanding the relationship between virus strain and severity; (v) providing candidate viruses for vaccine production; and (vi) providing information for vaccine virus selection.^17^

In the 2011 consultation, to strengthen surveillance standards, WHO reviewed the relevant evidence from research studies and the recommendations of technical experts conducting influenza surveillance. To meet WHO’s recommendations, the sensitivity and specificity of the case definition of influenza needed to be high and the language used needed to be simple enough to remove any ambiguity in the interpretation of the criteria.

## Process of change

### Influenza-like illness

In the guidelines to the 1999 case definition, clinical influenza was defined as “a sudden onset of fever, a temperature >38°C and cough or sore throat in the absence of another diagnosis”.^9^ When this definition was used, the occurrence of ILI in a community generally correlated with the seasonal levels of transmission of influenza virus.^10^ However, the sensitivity of the definition was generally only about 60% and its specificity ranged from 0%, e.g. when there was little circulation of influenza virus, to 60–90%, e.g. during each main influenza season and the 2009–2010 influenza pandemic.^11^^,^^12^ In studies conducted before WHO’s 2011 consultation, the most predictive symptoms of influenza were found to be cough, fatigue, fever and myalgia.^11^^–^^14^^,^^18^^,^^19^ As sore throat had been identified as a negative predictor of influenza^13^^,^^17^^,^^19^ and been found hard to diagnose in young children, it was omitted from the clinical case definition of ILI proposed in 2011.^17^ The criterion “absence of another diagnosis” was also omitted because its inclusion in the 1999 definition had resulted in the exclusion of ILI cases with underlying chronic conditions, e.g. asthma and congestive heart failure, that can influence influenza risk. The criterion “sudden onset of fever” was changed to “acute respiratory illness” in order to capture a more general syndrome. A time frame was specified to distinguish between old and new symptoms and to capture acute disease onset. The initial proposal was for a time frame of seven days from symptom onset^17^ but this was subsequently revised to 10 days to match the time frame for SARI. Finally, the measured fever component of the definition was changed from “>38°C” to “≥ 38°C” to account for the physicians and record-keepers who would round down slightly higher temperatures to 38°C. The proposed clinical case definition of ILI therefore became “an acute respiratory illness with a measured temperature of ≥ 38 °C and cough, with onset within the past 10 days”.

The proposed changes to the ILI case definition should result in enhanced specificity without greatly compromising the definition’s sensitivity (Box 1). These changes allow for a better alignment of the case definition of ILI with that of SARI. They also clarify some aspects of the previous ILI case definition that could be misinterpreted by health-care providers, including those working in sentinel sites and national influenza centres and influenza epidemiological unit surveillance officers.

Box 1 **Revision^a^ of clinical case definition for influenza-like illness, 2011**

**Omit “sore throat”**

Rationale: Studies have shown a lack of association between a sore throat and influenza in individuals with respiratory disease. Sore throat is also difficult to assess in infants.

**Omit “absence of another diagnosis”**

Rationale: The change prevents the exclusion of cases with underlying chronic conditions, such as asthma or chronic lung disease, which are risk factors for severe influenza.

**Change “sudden onset of fever” to “acute respiratory illness”**

Rationale: New term encompasses a broad, well-known syndrome and relates the definition to a clinically recognized condition.

**Add a timeframe, of ten days from symptom onset**

Rationale: Helps capture acute illness and distinguish new illness superimposed on old.

**Temperature of measured fever changed from “> 38 °C” to “≥ 38 °C”**

Rationale: Captures individuals with temperatures that, although above 38 °C, are recorded as 38 °C by clinicians and record-keepers who round down such temperatures.

**Result**

“An acute respiratory illness with a measured temperature of ≥ 38 °C and cough, with onset within the past 10 days”.

^a^ Compared with corresponding definition published in 1999.^9^

### Severe acute respiratory infection

Before 2011, a global-surveillance case definition of SARI did not exist. Three WHO Regions had independently developed their own definitions before the 2009–2010 influenza pandemic. These definitions were based on the case definition published by the Pan American Health Organization in 2005,^20^ which, in turn, was based on criteria set, as part of the integrated management of childhood illness strategy, for pneumonia and severe pneumonia (Box 2). For children younger than five years, the strategy’s criteria for pneumonia were considered relatively nonspecific for radiographically confirmed pneumonia. They were considered acceptable for use in the definition of SARI as they would capture both pneumonia caused by influenza and other respiratory diseases related to influenza, e.g. an exacerbation of asthma triggered by influenza and requiring hospitalization.^21^ By 2011, the definitions developed by the integrated management of childhood illness strategy were already widely in use and could be adapted to cover hospital-based influenza, i.e. the severe end of the influenza-related disease spectrum, including those patients that did not have associated pneumonia. Severe influenza without pneumonia represents a substantial proportion of the burden posed by hospitalized influenza patients.^21^^,^^22^

Box 2 **Criteria previously used in regional definitions of severe acute respiratory infection**

Among individuals aged above five years, the criteria used were those for pneumonia in this age group, i.e. sudden onset of fever above 38°C, cough or sore throat, shortness of breath or difficulty breathing, and requiring hospitalization.

Among individuals aged between two months and five years, the criteria used were either those for pneumonia in this age group, i.e. cough or difficulty breathing and a breathing rate above 60, 50 and 40 breaths per minute for those younger than two months, between two and 12 months and older than 12 months, respectively, or those for severe pneumonia in this age group, i.e. cough or difficulty breathing, requiring hospitalization and showing at least one of five danger signs.^a^

^a^ The five danger signs were: unable to drink or breastfeed; vomiting everything ingested; having convulsions; being lethargic or unconscious; and showing chest indrawing or stridor while calm.

During the WHO’s 2011 consultation, the consensus among countries conducting surveillance of severe influenza was that it was desirable and most practical to have one definition of severe illness for all age groups.^17^ Although the integrated management of childhood illness strategy’s case definitions might have some use in this context, they are intended for clinical management in outpatient clinical settings^23^ and are generally not used by physicians in those hospitals where SARI surveillance is focused.

In 2011, in discussions involving the development of criteria for a case definition of SARI, concerns were raised about use of the pneumonia symptoms “shortness of breath” and “difficulty breathing”.^17^ Both of these symptoms are difficult to quantify and are often misinterpreted to include conditions such as nasal obstruction. Moreover, substantial shortness of breath only occurs as a late sign in the course of respiratory infections and inclusion of this symptom could therefore reduce the sensitivity of the definition.^24^

In the pneumonia case definition, the clinical sign “measured fever” was considered too restrictive for hospitalized patients, especially for capturing those patients who may have taken antipyretics to reduce fever, patients who do not present with fever, as often seen in adults, and those who have progressive illness from ILI. “Measured fever” was therefore changed to “measured fever … or history of fever”. This change aimed to improve the sensitivity of the definition among hospitalized patients and allow more potential influenza cases to be identified for laboratory testing, and so allow the capture of a larger proportion of the disease burden due to influenza viruses (Box 3).

Box 3 **Development^a^ of a new case definition of severe acute respiratory infection, 2011**

**Drop use of separate criteria for pneumonia and severe pneumonia among children aged < 5 years**

Rationale: Use of a single SARI case definition for all age groups simplifies implementation. The IMCI case definitions represent a clinical management tool and not very suitable for surveillance.

**Drop “shortness of breath” and “difficulty breathing”**

Rationale: These symptoms are not sufficiently specific, do not add to the accuracy of the definition and are often misinterpreted.

**Add “history of fever”**

Rationale: This allows for the inclusion of cases that have taken antipyretics that could have reduced fever and those, such as many older adults and the chronically ill, that do not have fever when admitted.

**Increase time frame between symptom onset and presentation from seven days to 10 days**

Rationale: SARI cases that present between seven and 10 days after symptom onset appear no less likely to be laboratory-test-positive for influenza as those that present closer to symptom onset. Increasing the time frame to 10 days should therefore allow more cases to be captured, without markedly increasing the use of resources.

SARI: severe acute respiratory infection.^a^ Compared with previous regional definitions based on the criteria used by the Integrated Management of Childhood Illness strategy (Box 2).

Lastly, as with the most recent version of the ILI definition, “onset within the past 10 days” was added to the most recent version of the SARI definition (Box 3). Although a period of seven days was originally considered,^17^ a review of available data indicated that patients with SARI that presented between seven and 10 days after symptom onset were no less likely to be laboratory-test-positive for influenza, as those that presented closer to symptom onset (E Dueger, United States Centers for Disease Control and Prevention, unpublished data, 2013; MT Olivera da Costa, Belo Horizonte Municipal Health Secretariat, unpublished data, 2013).^24^^,^^25^ Extending the period to include patients within 10 days of symptom onset allowed more influenza cases to be captured, without sacrificing specificity. The proposed clinical case definition of SARI, among all age groups, therefore became “an acute respiratory illness with a history of fever or measured fever of ≥ 38 °C and cough, with onset within the past 10 days, requiring hospitalization”. To simplify the implementation process, the same criterion, i.e. “onset within the past 10 days”, was subsequently used in the case definitions of both ILI and SARI.

## Practical implications

Since the WHO’s recommendations for the revision of the case definition of ILI,^17^ several studies have examined the best predictors of influenza virus infections that are appropriate for all age groups. The results of these studies supported the use of the simplified criteria of “cough” and “fever” in, and the removal of “sore throat” from, the clinical case definition of such infections.^26^^–^^30^ When the performance of the 2011 WHO case definition of ILI was compared with that of three corresponding definitions, one developed by the European Centre for Disease Prevention and Control, one developed by the United States Centers for Disease Control and Prevention and one developed in Taiwan, China, the WHO definition was found to have the highest specificity.^28^^,^^30^

In a study conducted among hospitalized patients in rural India, addition of reported “history of fever” to a case definition of ILI that already included “measured fever”, improved sensitivity, but reduced specificity.^29^ Without “history of fever”, the definition gave poor sensitivity, particularly among patients younger than five years. Along with cough, measured and/or reported fever were considered to provide the best balance between sensitivity and specificity among the study population.^29^

In a Ghanaian study, performance of the 2011 WHO case definition of SARI was evaluated in both young and old hospitalized patients. In this study, 98% of the influenza cases with SARI presented with cough and current fever and/or history of fever.^31^ The accuracy of influenza identification was investigated among hospitalized patients in Kenya using case definitions of both SARI and pneumonia.^32^ Compared with the pneumonia definition, the SARI case definition gave higher sensitivity and lower specificity when applied to patients younger than five years and it gave higher specificity when applied to older patients. Since, for influenza surveillance, the performance of the pneumonia definition appeared generally similar to that of the SARI definition, it was suggested that hospital-based influenza surveillance could be integrated within the existing platforms for pneumonia surveillance.^32^

As the separation of influenza from other respiratory virus infections remains a challenge, any attempt at influenza identification that is based on a so-called one-size-fits-all approach will have its shortcomings.^33^ This is especially true when estimating the burden posed by influenza in all age groups. Although fever has been shown to be the one of the most predictive symptoms of influenza, it occurs in many other common respiratory diseases, particularly among young children.^28^ By expanding the SARI case definition to include history of fever, more young children infected with non-influenza respiratory viruses may be mis-identified as cases of influenza. As young children infected with influenza viruses do not present with some of the symptoms that, in adults, are useful clinical predictors of such infection, the use of the latest case definition of SARI for influenza surveillance among such children needs to be very cautious.^34^ This is particularly true during the peak in the influenza season, which tends to coincide with a peak in the circulation of other respiratory viruses. For the detection of infections with influenza viruses in children younger than five years, it may be advisable to use a tailored case definition, e.g. one that uses a higher temperature cut-off for fever^28^ or includes rhinorrhea or other additional respiratory symptoms^35^, to increase specificity.

The age-specific differences observed in the distributions of influenza virus subtypes^28^ may be only partially responsible for the age-related differences observed in health-seeking behaviour, following infection with an influenza virus.^36^ Such variation and the issue of infections with non-influenza respiratory viruses being confused with influenza, may lead to substantial sampling bias in attempts to follow influenza epidemiology on a national scale. Infections with respiratory syncytial virus have been shown to decrease the performance of a WHO clinical case definition when that definition was used for influenza surveillance.^34^ One aim of a global surveillance scheme for respiratory syncytial virus that is being developed by WHO is to assess the potential adverse impact of the virus on existing ILI and SARI surveillance.^37^

The primary goal of influenza surveillance is not to capture every influenza case, but to follow patterns in transmission and disease burden and changes in the influenza viruses that are circulating. Although WHO recommends the use of the ILI and SARI case definitions that resulted from the 2011 consultations and were finalized in late 2011, it does not necessarily advise against the use of other definitions, so long as they are broader and do not screen out any individuals who would otherwise have been screened in. Now more than ever, the pandemic threat posed by novel respiratory viruses underscores the need for a robust global system of influenza surveillance, based on global case definitions.
